# Non-Linear Optical Flow Cytometry Using a Scanned, Bessel Beam Light-Sheet

**DOI:** 10.1038/srep10751

**Published:** 2015-05-29

**Authors:** Bradley B. Collier, Samir Awasthi, Deborah K. Lieu, James W. Chan

**Affiliations:** 1 Center for Biophotonics, University of California, Davis; 2 Department of Biomedical Engineering, University of California, Davis; 3 Department of Internal Medicine, University of California, Davis; 4 Department of Pathology and Laboratory Medicine, University of California, Davis.

## Abstract

Modern flow cytometry instruments have become vital tools for high-throughput analysis of single cells. However, as issues with the cellular labeling techniques often used in flow cytometry have become more of a concern, the development of label-free modalities for cellular analysis is increasingly desired. Non-linear optical phenomena (NLO) are of growing interest for label-free analysis because of the ability to measure the intrinsic optical response of biomolecules found in cells. We demonstrate that a light-sheet consisting of a scanned Bessel beam is an optimal excitation geometry for efficiently generating NLO signals in a microfluidic environment. The balance of photon density and cross-sectional area provided by the light-sheet allowed significantly larger two-photon fluorescence intensities to be measured in a model polystyrene microparticle system compared to measurements made using other excitation focal geometries, including a relaxed Gaussian excitation beam often used in conventional flow cytometers.

Over the last few decades, flow cytometry has become an invaluable tool in the biomedical field; clinical diagnostics, therapeutics, cell biology, and many other fields benefit from its cellular analysis and sorting capabilities^1^,^2^,^3^,^4^,^5^,^6^,^7^,^8^. These functions are performed at a high throughput (>10,000 cells/second) by optically analyzing individual cells in a microfluidic environment. Cells (or particles) of interest are suspended in solution and hydrodynamically focused into a narrow stream to allow cells to pass through an analysis region one at a time^3^,^8^. Once analysis of the cells has been performed, cells can be sorted from complex mixtures into pure populations using a variety of methods^1^,^2^,^4^,^9^,^10^,^11^.

Although cellular analysis can be performed using an assortment of techniques (*e.g*. optical, electrical, acoustic), sorting of cells into pure populations is often performed using fluorescence activated cell sorting (FACS). This process typically requires conjugation of a fluorescent label using antibodies specific to receptors or biomarkers representative of the cellular phenotype of interest^2^,^3^,^12^. However, these labels are not ideal in some cases due to nonspecific binding, limitations in the availability of conjugation chemistries, and conjugation times^8^,^13^,^14^. The use of animal-derived antibodies for labeling the cells may also be a concern of the FDA for *in vivo* applications, since exposure of the cells to animal origin products may increase the risk of non-human pathogen transmission (e.g. viruses, prions)^15^. In addition, the presence of the labels can cause disruption of normal cellular function or even cell death^16^,^17^. For these reasons, cellular labeling may be acceptable for *ex vivo* applications such as rare cell detection for disease diagnosis, but for cells intended for research applications or *in vivo* use (*i.e*. transplantation into the body), new techniques of purification need to be explored. This is especially true of the growing field of regenerative medicine where inefficient differentiation of stem cells into specific cell lineages requires cells to be sorted prior to therapeutic use in order to eliminate any undesired cell types or potentially tumorigenic undifferentiated pluripotent stem cells^13^,^18^.

To overcome the issues with using exogenous labels, label-free techniques are an attractive alternative to fluorescence methods for recognizing cells of interest without being detrimental to cellular function or health^2^,^19^. Non-linear optical phenomena or NLO (*e.g*. second harmonic generation, two-photon autofluorescence, coherent anti-Stokes Raman scattering) are of growing interest for label-free analysis because of the ability to measure the intrinsic optical response of biomolecules found in cells^12^,^16^,^17^,^18^,^20^,^21^,^22^,^23^,^24^. For example, we have previously reported on an intrinsic second harmonic generation signal that could potentially be used as a specific marker for label-free, non-genetic purification of stem cell derived cardiomyocytes.^13^ Generation of non-linear signals, however, requires higher energy or photon density in the excitation beam compared to traditional linear optical phenomena such as fluorescence. The ellipsoid-shaped, relaxed Gaussian beams that are traditionally used for flow cytometry^3^,^8^ may not be sufficient for NLO phenomena because of the large drop in photon density with such a relaxed beam focus. A tightly focused Gaussian beam, which is typically used for NLO imaging applications and has been demonstrated for NLO signal generation in a flow cytometry configuration^17^,^21^, can be used but the small focal volume relative to typical cell diameters (~10 μm) and the cross section of the sample stream, will mean that the entire cell volume will not be excited or cell events may be missed entirely as cells flow past the excitation region without entering the small interrogation volume of the excitation beam. Scanning of the tightly focused laser beam has also been demonstrated for NLO flow cytometry^12^,^16^,^20^, and although this will allow excitation of a greater volume of each individual particle, it may still not allow excitation of optical signals in the entire volume of each particle and events may still be missed.

In this study, we propose that an appropriate optical configuration needs to achieve a balance between high photon densities and a large excitation area in order to optimize the excitation efficiency for NLO flow cytometry. To achieve this balance, a novel light-sheet excitation method was investigated for generating NLO signals in a microfluidic environment. Similar to light-sheets used for imaging studies^25^,^26^,^27^, the light-sheet was constructed using a scanned Bessel beam. The reconstructive nature of the Bessel beam allows the excitation light to be focused into a narrow cylinder which when scanned effectively covers the cross-section of the sample channel while maintaining the high photon density for efficient generation of NLO signals. The scope of this work is to compare the NLO excitation efficiency of this light-sheet method to different excitation geometries. This was achieved using two-photon fluorescence (2PF) from fluorescent polystyrene particles as a model system similar to the use of compensation beads for the calibration of flow cytometers.

## Results and Discussion

### Bessel Beam Characterization

**Figure 1:**
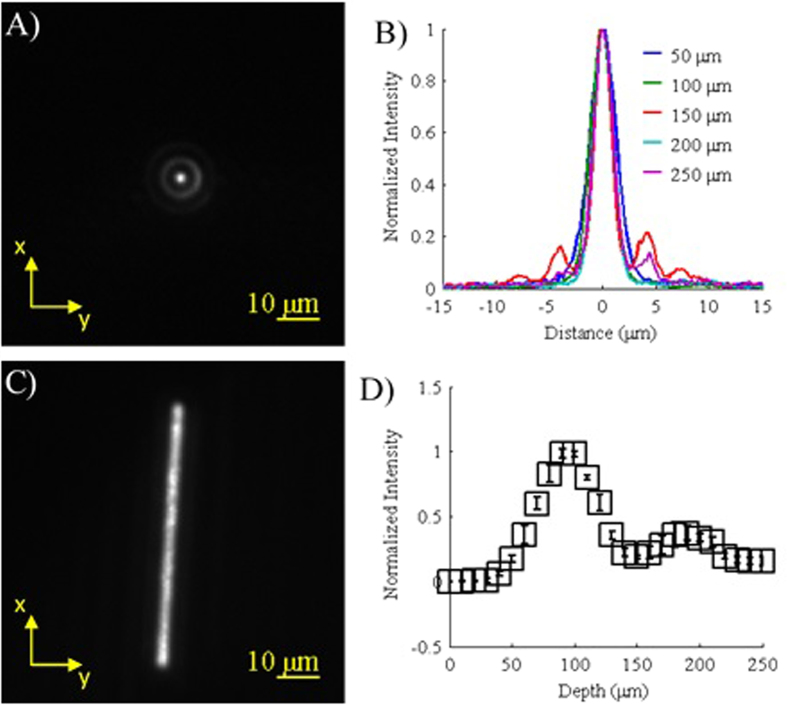
Characterization of the Bessel beam. (**A**) Example of a Bessel beam profile obtained using a QD film (z = 150 μm). (**B**) Normalized plots of the Bessel beam profile for various depths along the z-axis. (**C**) Image of the Bessel beam being scanned where the exposure time is much longer than the scanning period. (**D**) Relative intensities of the Bessel beam along the z-axis (*n* = 5, error bars represent standard deviation).

The images and data obtained for characterization of the Bessel beam can be seen in Fig. 1. As expected, focusing of the annulus resulted in a Bessel beam with a strong central maximum with a width (FWHM) of 2.26 μm and was surrounded by rings of lesser intensity (Fig. 1A,B)^25^. Similar to previous reports, the beam’s intensity along the z-axis also showed a Gaussian-like profile with a depth (FWHM) of 59.8 μm (Fig. 1D)^25^. The second weaker peak visible near 175 μm is most likely due to imperfections in the annulus where fainter rings are visible inside of the primary annulus (Fig. S1). When scanned at ±1 V, the Bessel beam was visible over a distance greater than 56 μm (Fig. 1C) which is sufficient to cover the width (33.3 μm) of the sample stream in the microfluidic channel. It should also be noted that the scanning speed of the mirror (1 kHz) combined with the width of the Bessel beam (2.26 μm) allows the entire volume of the particles to be analyzed as they flow through the microfluidic channel at 1.7E3 μm/s (see Methods). For faster flow rates, a higher scanning frequency will be required in order to ensure that the entire volume of the particle is analyzed.

### Gaussian Beam Characterization

**Figure 2:**
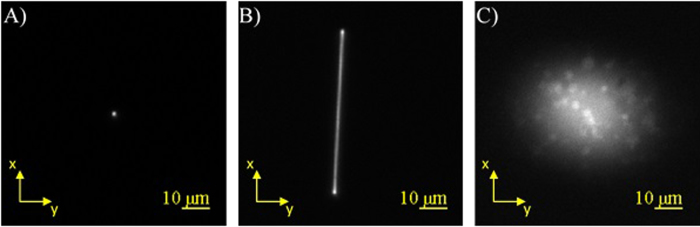
Characterization of the tightly-focused and relaxed Gaussian beams. (**A**) Normalized image of fluorescence from a QD film showing the beam profile of a tightly focused Gaussian beam. (**B**) Normalized image of the tightly focused Gaussian beam being scanned across a QD film. (**C**) Normalized image of a relaxed Gaussian beam exciting the same QD film. Spots in the image are due to imperfections in the QD film.

The Gaussian excitation systems showed beam profiles that were also similar to the expected profiles (Fig. 2). The tightly focused Gaussian (Fig. 2A) had a beam width (FWHM) of 1.07 μm and when scanned displayed a similar width along the x-axis as the scanned Bessel beam (Fig. 2B). The relaxed Gaussian (Fig. 2C) displayed an average width (FWHM) of 20.7 μm. Increasing the beam width further to match the width of the sample stream was not investigated because reducing the photon density further would have led to a further decrease in the 2PF signal.

### Microfluidic Measurements

**Figure 3:**
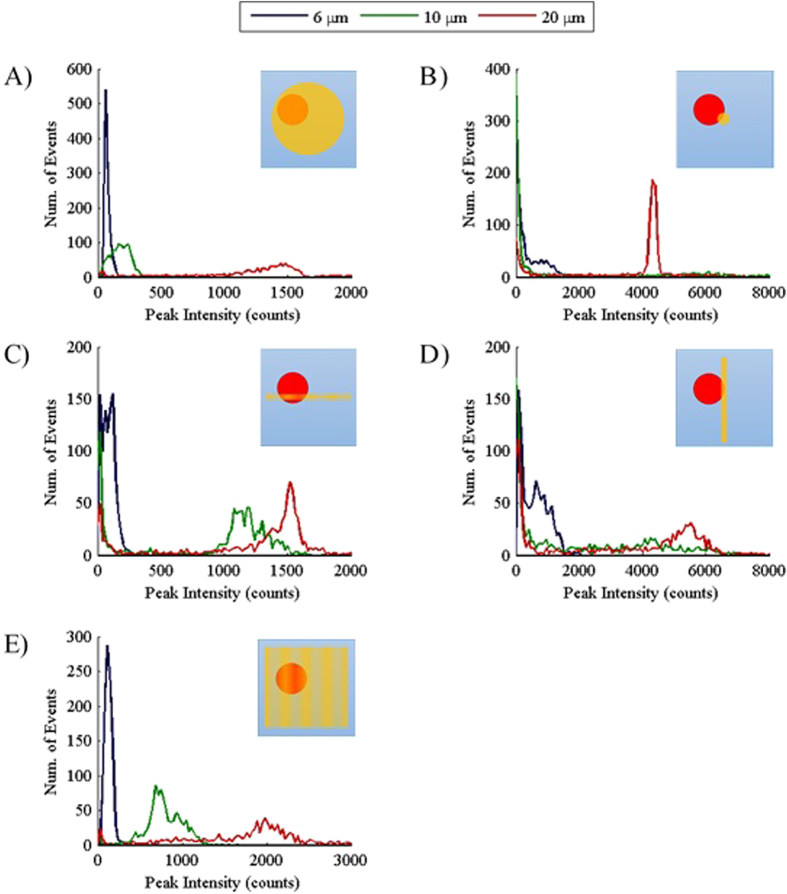
Distribution plots of the peak intensities for each particle size for different excitation beam profiles. (**A–E**) Graphs showing the distribution of the measured 2PF peak intensities (*n* = 1000) for each particle size (legend at top) and different excitation beams: (**A**) relaxed Gaussian, (**B**) tightly focused Gaussian, (**C**) scanned Gaussian, (**D**) static Bessel beam, (**E**) scanned Bessel beam or light-sheet. A few peaks with higher intensity were recorded for each graph but were not shown because they are most likely the result of aggregate measurements and were far less frequent than the peak intensities representative of the expected distributions for each particle size. In the upper right hand corner of each graph is a generalized cross-section of the sample stream (xz-plane) showing a depiction of a particle (red) flowing through each excitation beam (yellow) used to obtain the data shown in the graph. The beams in (**C**) and (**E**) do not have solid fills to represent that the beam is being scanned.

To analyze the data obtained using the different excitation beams, the peak intensity of each measured 2PF event was plotted in a distribution similar to a histogram (Fig. 3). The relaxed Gaussian excitation geometry produced distribution plots with well-defined peaks (*i.e*. minimal data skew) due to the effective coverage of the cross-sectional area of the sample stream in the *xz*-plane (Fig. 3A) by the laser beam. This coverage allows the entire volume of each particle to be interrogated, leading to the reproducible detection of 2PF signals. The broadness of the distributions increases with particle size due to the increasing variability in the amount of dye loaded into each particle (Fig. S2A).

Despite the lower input power (see Methods), the higher photon density in the tightly focused Gaussian beam, which is typically used in NLO microscopy, produced higher peak intensities than excitation using the relaxed Gaussian (Fig. 3B). For example, the 20 μm distribution increased from approximately 1500 counts to over 4000 counts. However, the distributions were not well-defined as the peak intensities were skewed towards lower peak intensities where many events were recorded for each particle size. This artifact is a result of the inefficient interrogation of the particles. The small excitation focal volume of the tight Gaussian, relative to the particle size, does not effectively cover the cross-sectional area of the sample stream and allows particles to pass that are only weakly excited (and possibly not excited at all) because inadequate coverage of the microfluidic channel by the excitation beam. The 20 μm particles produced a more well-defined peak than the other sizes because the larger size increases the probability of reproducible excitation. It is also important to note that the small size of the beam relative to the particles allows only dye along a narrow line or one dimension (1D) within the particle volume to be excited as the particle flows through the tightly focused Gaussian beam. Evidence of this reduced excitation volume can be seen in the 10 μm distribution. Although the 20 μm particles have a larger total amount of dye present, many 10 μm events had a higher measured peak intensity (see Fig. S3 for a more detailed view of this data) due to their higher dye concentration (Fig. S2B). This was not the case for any other excitation beam profile that was utilized.

Scanning the tightly focused Gaussian along the *x*-axis increases the excitation volume as well as the probability of the particles hitting the beam. This led to an improvement in the delineation of the peak distributions compared to the static tightly focused Gaussian beam results (Fig. 3C). However, a data artifact was still present as the distributions were again skewed towards lower peak intensities for all particle sizes. In addition, the peak intensities for each particle were reduced due to the reduced time the particles spent in the excitation beam as a result of scanning over the entire width of the sample stream. As expected, the 10 μm distribution shifted back to the left of the 20 μm distribution due to the addition of a second dimension (2D) of interrogation. This allows more dye to be excited in the larger particles as interrogation is now performed through a plane of the particle rather than along a line. Excitation using a static Bessel beam produced distributions similar to the scanned Gaussian excitation because it also utilizes 2D interrogation of the particles (Fig. 3D). Differences in the peak intensity are due to the differences in the excitation power used (see Methods). Also, the apparent increase in the baseline of the distributions of the static Bessel beam compared to the scanned Gaussian is due to the focusing of the collection objective. Although both of these excitation modalities do not provide effective coverage of the channel, the scanned Gaussian is always on the same plane as the focus of the collection objective. This leads to efficient measurement of 2PF when a particle hits the excitation beam or measurement of low peak intensities because the particle does not hit the beam. Because the static Bessel beam is perpendicular to the focal plan of the collection objective, particles can hit the beam and 2PF events will not be as effectively measured which results in measured peak intensities between the expected peak of the distribution and the data skewed towards lower peak intensities.

**Figure 4:**
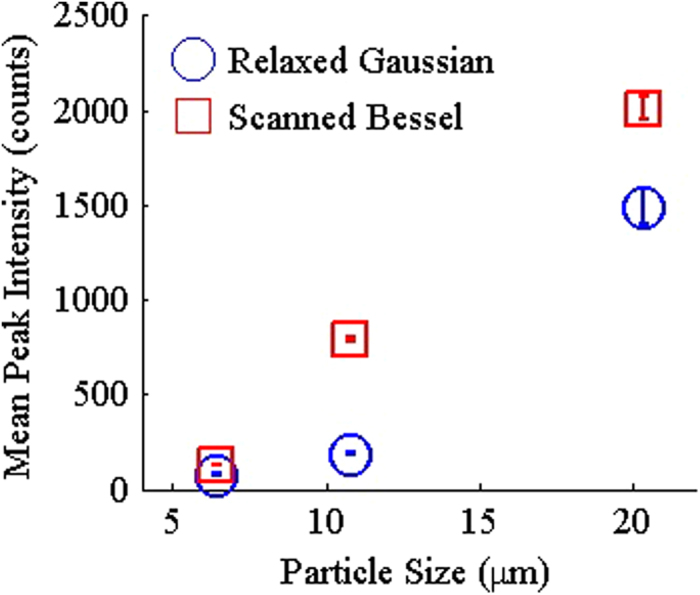
Comparison of the results for the relaxed Gaussian and scanned Bessel excitation beams. Mean intensities for each particle size measured are shown for 3D excitation methods tested. Error bars represent 95% confidence interval with *n* = 1000.

By scanning the Bessel beam, we can achieve better 3D excitation of the particles allowing the entire volume of the particles to be interrogated. This allow interrogation similar to excitation using the relaxed Gaussian but with a higher photon density. The peak intensity distributions obtained were well-defined, similar to results obtained from relaxed Gaussian measurements (Fig. 3E). In addition, the distributions for each particle size showed minimal overlap and higher peak intensities compared to results from the relaxed Gaussian with the same input power (Fig. 4). Overall, the distributions obtained for the light-sheet more closely matched the measured amount of dye in the particles (Fig. S2A) than any other excitation modalities tested. Similar to the results with the tightly focused Gaussian beam, scanning of the Bessel beam resulted in lower peak intensities compared to the static Bessel Beam results. Nonetheless, these results demonstrate that a scanned Bessel beam can be used to achieve a greater photon density and hence more efficient excitation of NLO than a relaxed Gaussian beam while still providing effective coverage of the cross-sectional area of the sample stream.

It should also be noted that ellipsoid-shaped beams, which are often utilized in commercial flow cytometers for traditional linear optical measurements, will also increase the photon density over the circular-shaped, relaxed Gaussian demonstrated. By compressing the energy of the relaxed Gaussian along the direction of particle flow (the *y*-axis in Fig. 2C), the efficiency of NLO excitation can be slightly improved. Cylindrical lenses have also been utilized to create compressed excitation beams for measuring (one-photon) fluorescence in flow cytometry applications^28^,^29^,^30^. Although these approaches allow higher photon density in the excitation beam, the measured intensities are expected to be higher for the light-sheet demonstrated due to the higher photon density in the Bessel beam. This higher photon density afforded by a light-sheet compared to these alternatives may be particularly important for weak NLO phenomena, such as what we have previously observed in probing the second harmonically generated signals from immature stem cell derived cardiomyocytes^13^. In addition, the Bessel beam light-sheet is expected to have a more uniform intensity along the *x*-axis due to scanning of the beam while ellipsoid-shaped beams or beams made using a cylindrical lens will still display a Gaussian-shaped intensity profile along the *x*-axis. However, a system utilizing a cylindrical lens or a linear diffuser to create a light-sheet for generation of NLO signals still warrant future investigation as it may be advantageous in some cases due to cost and simplicity if a greater photon density is not required.

In summary, a novel light-sheet excitation system for the excitation of NLO was compared to several optical excitation approaches similar to those reported for traditional (relaxed Gaussian) and NLO flow cytometry applications (scanned and static tightly focused Gaussian). These systems explored the trade-off between photon density and excitation area. Although tighter focusing of the beam led to an increase in the measured peak intensities of the NLO signal, distinct distributions could not be obtained as a result of the reduced probability of particles hitting the relatively small excitation beam. In order to obtain distinct intensity distributions and efficiently generate NLO, the excitation beam must provide effective coverage of the cross-sectional area of the sample stream allowing the entire volume of each particle to be interrogated as was demonstrated with a relaxed Gaussian excitation and a light-sheet system consisting of a scanned Bessel beam. Although both of these excitation approaches were able to measure well-defined distributions for each particle size, the light-sheet system was able to produce higher peak intensities and showed better distinction between the different distributions due to the higher photon density in the beam. Even though throughput of the system was not investigated within the scope of this work, implementation of faster electronics (*e.g*. increased frequency of the scanning mirror) will allow analysis of thousands of cells per second similar to traditional flow cytometers. In addition, faster electronics will allow an even more thorough interrogation of the particle volume. In the future, this measurement system will be combined with a sorting mechanism to perform purification of stem cell derived cardiomyocytes through second harmonic generation of light interacting with the myosin domains of the cardiomyocytes.

## Methods

**Figure 5:**
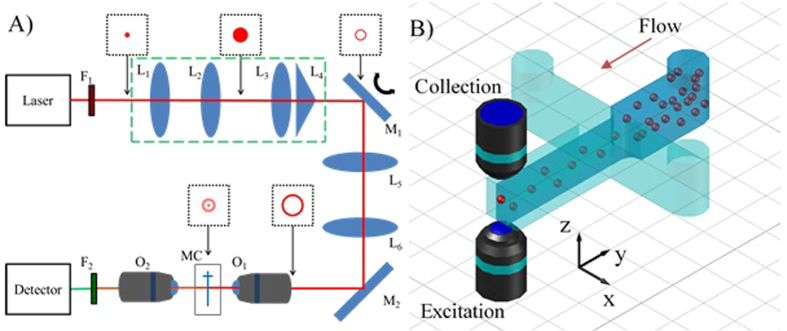
Schematic layout and diagram of the optical and microfluidic parts of the system. (**A**) Generalized diagram of the light-sheet and Gaussian systems used for excitation and collection of two-photon fluorescence from polystyrene particles. The lenses in the green dashed box shape the beam into an annulus and were removed for the tightly focused Gaussian system. The images in the black boxes provide a general depection of the beam mode at that location in the Bessel beam system. Further description of the filters (F) lenses (L) mirrors (M) and objectives (O) used can be found in Table S1. (**B**) Diagram of the microfluidic channel used to hydrodynamically focus the sample stream into the region between the excitation and collection objectives using two buffer channels on either side of the sample. Direction of flow is indicated by the red arrow.

A light-sheet system was built similar to those previously reported for imaging applications^25^,^26^,^27^,^30^,^31^. A Taccor C laser from Laser Quantum with a repetition rate of 1 GHz, a pulse width of 80 fs, and a wavelength of 920 nm was used as the excitation source. After expanding the beam (3X), an annulus was formed using a lens-axicon combination rather than an annular mask in order to conserve laser power^25^,^31^,^32^,^33^,^34^. The annulus was focused onto a mirror (Cambridge Technologies 8310 K) which was used in a static and a scanning mode where a 1 kHz triangle wave (±1 V) was used to control the speed and deviation of scanning. The annulus was then imaged onto the back of an excitation objective (60X, NA = 1.2) using a 2X beam expander. When focused, the annulus forms a Bessel beam where the dimensions of the beam are dependent upon the diameter and thickness of the annulus^25^. A *t*-shaped microfluidic channel (LabSmith 02-0768-0106-05) with a channel width and height of 100 μm was held between the excitation and collection objectives using a custom sample holder. Two-photon fluorescence (2PF) events from the samples were collected in the trans-direction using a long working distance objective (50X, NA = 0.55) and focused onto a photon counting PMT (Hamamatsu H7421-40) using a lens. Data sampling was performed at 1 kHz which allowed one measurement to be obtained for one complete scan of the channel (*i.e*. one period of the triangle wave). Due to the integrative nature of the photon counting PMT, faster sampling rates are not necessary and may cause oscillations in the 2PF events due to the scanning of the mirror (see Fig. S4). Control of this system was performed using a DAQ board (National Instruments USB-6259), a function generator, and a computer running custom LabVIEW software. A diagram of the optical components of the system can be seen in Fig. 5A.

The results from the light-sheet system were compared to two Gaussian systems. The first consisted of a tightly focused Gaussian beam created using the scanning mirror and 2X beam expander of the light-sheet system. This led to a beam that slightly under-filled the excitation objective. Similar to the light-sheet system, this system was also used in both a scanning and static mode. A relaxed Gaussian system with a larger beam waist was also created by focusing the laser beam directly onto the microfluidic channel using only a long focal length lens (f = 250 mm). To control the power of the excitation beam, a variable attenuator (consisting of a 1/2 wave plate and a polarizer) was part of each system. For the relaxed Gaussian and Bessel beam excitation, the laser power was set to 100 mW. Less power (16.7 mW) was needed for the experiments using the tightly focused Gaussian because NLO signals are more easily generated due to the high photon density in the beam at the focus compared to the other methods of excitation. A list of the optical components used in all systems can be found in Table S1.

To characterize the different beams, a quantum dot (QD) film was created by drying water-soluble QDs with an emission wavelength near 600 nm (NN-Labs, CZW-O-1) on a glass coverslip. This allowed the beam width, Bessel beam length, and distance scanned to be determined. The annulus was imaged directly onto a CCD (Roper Scientific Photometrics CoolSNAP ES) after reducing the beam power. In order to test the effectiveness of each system, 2PF from polystyrene particles labeled with Nile Red was measured. Three sizes of microparticles tested were 6.42, 10.8, and 20.3 μm (Spherotech FP-60560-02, FP-10056-2, FP-20056-5) and will be referred to as 6, 10, and 20 μm particles, respectively. Particles of each size were suspended in 0.1% solutions of IGEPAL CA-630 (U.S. Biological, N3500) to help hold the particles in suspension and prevent aggregation. Three syringe pumps (New Era Pump Systems NE-3000) were each set to a flow rate of 20 μL/hr to hydrodynamically focus the sample stream to an approximate width of 33.3 μm leading to a particle velocity of 1.7E3 μm/s. The sheathing solutions used to focus the sample stream consisted of 0.01% IGEPAL CA-630. Figure 5B shows a diagram of the microfluidic channel used to control the sample flow and includes the orientation of the axes referenced herein.

Data obtained during microfluidic measurements was analyzed by subtracting the baseline of the raw data using a recursive linear fitting algorithm. An example of the baseline-subtracted data can be found in Fig. S5. The *findpeak* function of MATLAB was then used to determine the peak intensities of 2PF measurements. For this function, the minimum peak height was set to 10 times the standard deviation of the baseline to reduce the number of peaks due to signal noise and the minimum peak distance was set to 10 ms to reduce peaks where more than one particle (*e.g*. aggregates) were simultaneously measured.

## Additional Information

**How to cite this article**: Collier, B. B. *et al*. Non-Linear Optical Flow Cytometry Using a Scanned, Bessel Beam Light-Sheet. *Sci. Rep*. **5**, 10751; doi: 10.1038/srep10751 (2015).

## Supplementary Material

Supplementary Information
