# Supplementary material for: Non-Linear Optical Flow Cytometry Using a Scanned, Bessel Beam Light-Sheet
Source: Sci Rep. 2015 May 29;5:10751. doi: 10.1038/srep10751 (PMC4448227; doi:10.1038/srep10751)
Supplement: Supplementary Information [file srep10751-s1.pdf]

Non-Linear Optical Flow Cytometry Using a Scanned, Bessel Beam Light-Sheet

Supplementary Information

Bradley B. Collier[1], Samir Awasthi[1,2], Deborah K. Lieu[3], James W. Chan[1,4*]

[1]Center for Biophotonics, University of California, Davis
[2]Department of Biomedical Engineering, University of California, Davis
[3]Department of Internal Medicine, University of California, Davis
[4]Department of Pathology and Laboratory Medicine, University of California, Davis
[*]jwjchan@ucdavis.edu

Table S1. List of the parts used for Bessel beam (BB), tightly focused Gaussian (TG), and relaxed Gaussian (RG) systems. It is also important to note other optical elements that were utilized but are not depicted above. Specifically, several mirrors were utilized for beam alignment and a variable attenuator (consisting of a 1/2 wave plate and a polarizer) was used to control the laser beam power.

Label	Company	Model/Part #	Description	Function	System
Laser	Laser Quantum	Taccor C	1 GHz rep. rate, 80 fs pulse width, 920 nm wavelength	Sample excitation	All
F_1	Semrock	RazorEdge LP647	647 nm long pass filter	Laser clean-up	All
L_1	Thorlabs	AC127-025-B	Achromatic lens	3x Keplarian telescope	BB
L_2	Thorlabs	AC254-075-B	Achromatic lens	3x Keplarian telescope	BB
L_3	Thorlabs	AC254-075-B	Achromatic lens	Annulus focusing	BB
L_4	Thorlabs	AX2505-B	Axicon	Annulus formation	BB
M_1	Cambridge Technologies	8310K	Scanning mirror	Scan beam across sample	BB, TG
L_5	Thorlabs	AC254-100-B	Achromatic lens	2x Keplarian telescope	BB, TG
L_6	Thorlabs	AC254-200-B	Achromatic lens	2x Keplarian telescope	BB, TG
M_2	Thorlabs	PF10-03-P01	Silver mirror	Align beam onto objective	All
O_1	Olympus	UPlanApo/IR 60X/1.20W	60x water objective	Beam focusing onto sample	BB, TG
MC	Labsmith	02-0768-0106-05	t-shaped microfluidic channel	Hydrodynamically focus sample	All
O_2	Nikon	CFPlan 50X/0.55NA, ELWD	50x, extra long working distance objective	Signal collection	All
F_2	Chroma	HQ610SP	610 nm short pass filter	Eliminate any excitation light	All
Detector	Hamamatsu	H7421-20	Photon counting PMT	Measure fluorescence	All
--	Thorlabs	LA1461	Plano-convex lens, f=250mm	Relaxed Gaussian beam	RG

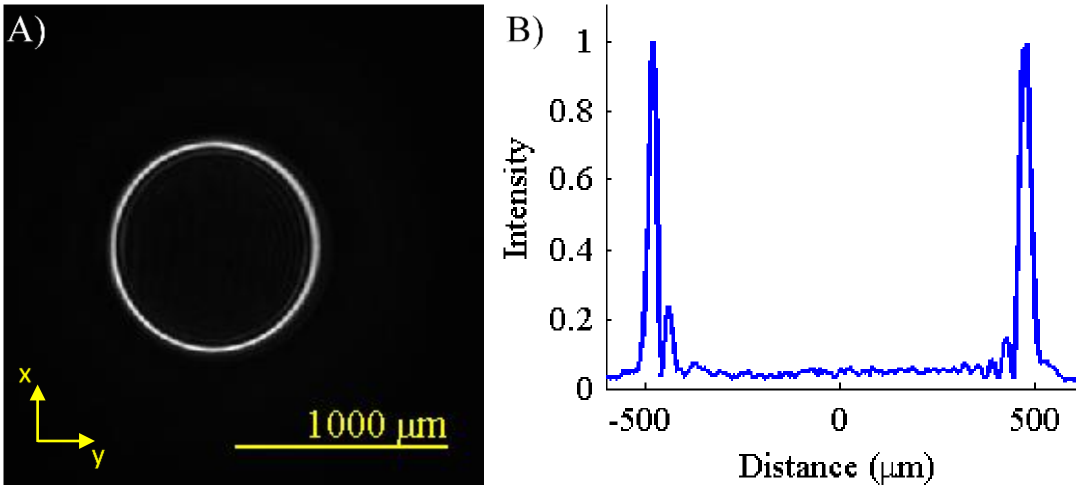

Figure S1. (A) Normalized image of the annulus obtained by reducing the laser power and capturing the annulus directly on a CCD. (B) Profile through the center of the annulus. The outer and inner diameters are 981.4 μm and 926.2 μm, respectively. The peak to peak width is 954.6 μm.

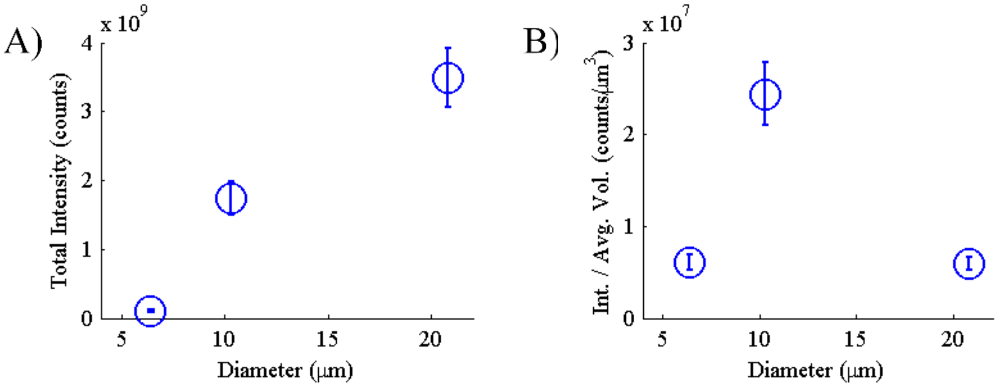

Figure S2. (A) The average total intensity for 6, 10, and 20 μm particles (*n*=25) displayed is representative of the total dye present in each particle. This data was obtained by summing z-stack images taken using a confocal microscope and one-photon fluorescence. (B) The relative dye concentration was also investigated by dividing the total intensity by the average particle volume. Although the total amount of dye is higher in the 20 μm particles, the 10 μm particles have a dye concentration that is approximately 4 times larger. Error bars represent standard deviation in both figures.

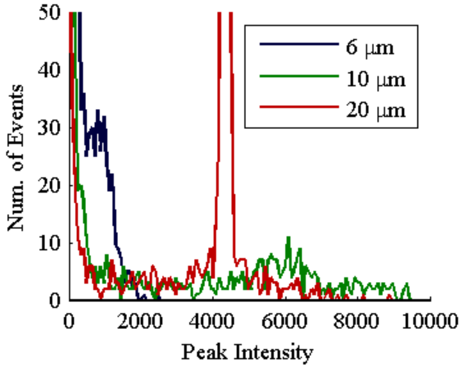

Figure S3. A zoom in of the data shown in **Error! Reference source not found.**B for excitation using a static, tightly focused Gaussian beam. Closer inspection shows the measured peak intensity for several 10 μm particles was larger than the peak intensities measured for 20 μm particles.

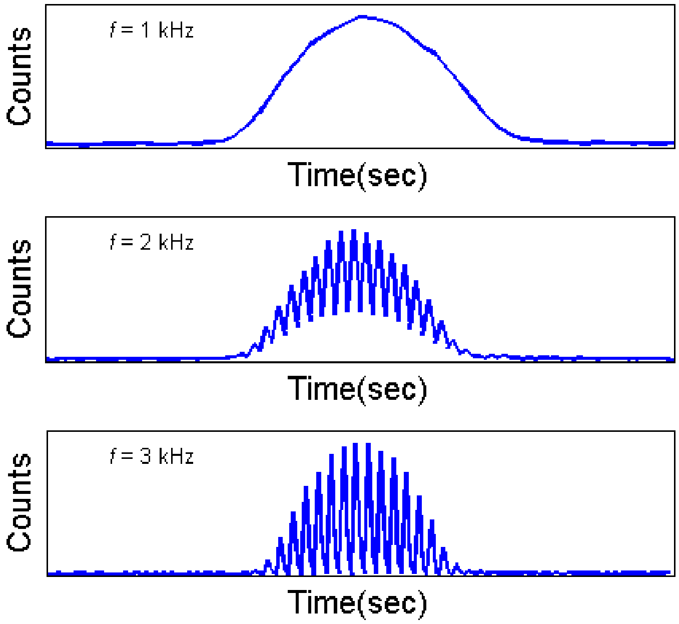

Figure S4. These graphs show the effect of increasing the sampling rate used to monitor the PMT. The top graph shows that when the sampling rate is set to 1 kHz (as was done for results in the manuscript), a smooth 2PF event can be obtained. However, as the sampling rate is increased, oscillations are seen in the 2PF events due to scanning of the beam off and on the particle. To obtain data that is more easily manageable a 1 kHz sampling rate was utilized which corresponds to 1 sample per period of the beam scan.

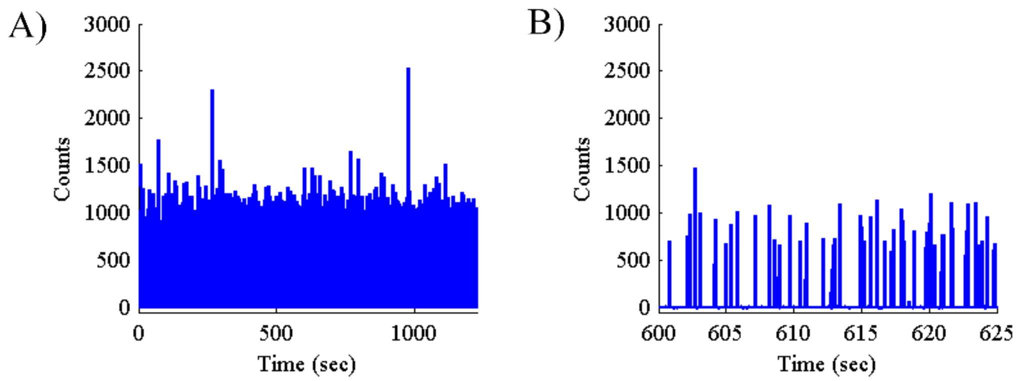

Figure S5. (A) Raw data of the 10 μm particles excited with a scanned Bessel beam. (B) A zoom in on the same set of data.
